# Exercise in Pregnancy and Children’s Cardiometabolic Risk Factors: a Systematic Review and Meta-Analysis

**DOI:** 10.1186/s40798-018-0148-x

**Published:** 2018-08-02

**Authors:** Laetitia Guillemette, Jacqueline L. Hay, D. Scott Kehler, Naomi C. Hamm, Christopher Oldfield, Jonathan M. McGavock, Todd A. Duhamel

**Affiliations:** 1 Children’s Hospital Research Institute of Manitoba, John Buhler Research Center, University of Manitoba, 511-715 McDermot Avenue, Winnipeg, MB R3E 3P4 Canada; 2 Health, Leisure & Human Performance Research Institute, Faculty of Kinesiology & Recreation Management, University of Manitoba, Winnipeg, MB Canada; 3 Institute of Cardiovascular Sciences, St. Boniface Hospital Research Centre, Winnipeg, MB Canada

**Keywords:** Developmental origins of health and disease, Birth weight, Prenatal exercise, Cardiometabolic health, Fat mass, Offspring

## Abstract

**Background:**

Maternal metabolic health during the prenatal period is an established determinant of cardiometabolic disease risk. Many studies have focused on poor offspring outcomes after exposure to poor maternal health, while few have systematically appraised the evidence surrounding the role of maternal exercise in decreasing this risk.

The aim of this study is to characterize and quantify the specific impact of prenatal exercise on children’s cardiometabolic health markers, at birth and in childhood.

**Methods:**

A systematic review of Scopus, MEDLINE, EMBASE, CENTRAL, CINAHL, and SPORTDiscus up to December 2017 was conducted.

Randomized controlled trials (RCTs) and prospective cohort studies of prenatal aerobic exercise and/or resistance training reporting eligible offspring outcomes were included.

Four reviewers independently identified eligible citations and extracted study-level data. The primary outcome was birth weight; secondary outcomes, specified a priori*,* included large-for-gestational age status, fat and lean mass, dyslipidemia, dysglycemia, and blood pressure. We included 73 of the 9804 citations initially identified. Data from RCTs was pooled using random effects models. Statistical heterogeneity was quantified using the *I*^2^ test. Analyses were done between June and December 2017 and the search was updated in December 2017.

**Results:**

Fifteen observational studies (*n* = 290,951 children) and 39 RCTs (*n* = 6875 children) were included. Observational studies were highly heterogeneous and had discrepant conclusions, but globally showed no clinically relevant effect of exercise on offspring outcomes. Meta-analyzed RCTs indicated that prenatal exercise did not significantly impact birth weight (mean difference [MD] − 22.1 g, 95% confidence interval [CI] − 51.5 to 7.3 g, *n* = 6766) or large-for-gestational age status (risk ratio 0.85, 95% CI 0.51 to 1.44, *n* = 937) compared to no exercise. Sub-group analyses showed that prenatal exercise reduced birth weight according to timing (starting after 20 weeks of gestation, MD − 84.3 g, 95% CI − 142.2, − 26.4 g, *n* = 1124), type of exercise (aerobic only, MD − 58.7 g, 95% CI − 109.7, − 7.8 g; *n* = 2058), pre-pregnancy activity status (previously inactive, MD − 34.8 g, 95% CI − 69.0, − 0.5 g; *n* = 2829), and exercise intensity (light to moderate intensity only, MD − 45.5 g, 95% CI − 82.4, − 8.6 g; *n* = 2651). Fat mass percentage at birth was not altered by prenatal exercise (0.19%, 95% CI − 0.27, 0.65%; *n* = 130); however, only two studies reported this outcome. Other outcomes were too scarcely reported to be meta-analyzed.

**Conclusions:**

Prenatal exercise does not causally impact birth weight, fat mass, or large-for-gestational-age status in a clinically relevant way. Longer follow up of offspring exposed to prenatal exercise is needed along with measures of relevant metabolic variables (e.g., fat and lean mass).

**Protocol Registration:**

Protocol registration number: CRD42015029163.

**Electronic supplementary material:**

The online version of this article (10.1186/s40798-018-0148-x) contains supplementary material, which is available to authorized users.

## Key Points

- In general, birth weight is not impacted in a clinically significant manner by prenatal exercise programs.
- Our understanding of the impact of prenatal exercise on the child’s metabolic health and future risk of cardiometabolic disease is limited as very few trials and cohort studies examined other health indicators aside from birth weight—an imperfect marker of health and future outcomes.
- Few trials and cohort studies followed up the children to see potential lasting effects of maternal prenatal exercise on their metabolic health.
- Clinicians looking to counsel their clients might highlight that while prenatal exercise is perfectly safe for the baby, the best evidence currently available indicate exercise is not sufficient by itself to protect the child against cardiometabolic diseases.

## Background

Obesity and metabolic syndrome are two of the most common chronic diseases among children [1–3]. Recent evidence suggests these conditions have their roots in utero as maternal obesity, dyslipidemia, and hyperglycemia are associated with child cardiometabolic health [4–9]. Animal- and population-based studies suggest that prenatal exposures may influence offspring development and cardiometabolic risk in childhood [10, 11]. Moderate-intensity exercise at least three times per week can maintain or improve maternal physical fitness [12] and cardiovascular health during pregnancy through a decrease in blood pressure [13], plasma triglycerides [13, 14], and insulin resistance [15–17]. Therefore, prenatal exercise could create a beneficial fetal milieu and reduce the risk of obesity and metabolic syndrome for the offspring by regulating weight and cardiometabolic factors at birth and later in childhood. However, comprehensive syntheses of high-quality evidence on this topic are scarce.

Previous systematic reviews examining the role of prenatal exercise on offspring outcomes have been conducted with heterogeneous results and the interpretation of their findings are limited for several reasons: (1) the primary outcome of interest for previous systematic reviews varied (birth weight or large-for-gestational age (LGA) status); (2) inclusion criteria were variable (types of exercise targeted; inclusion of diet as an intervention; type and number of databases queried; low quality study designs); (3) there were flaws in methodological rigor (type of analysis, pooling heterogeneous studies together); and (4) few evaluated health outcomes in offspring beyond birth weight. The reviews that found a reduction in birth weight (from − 440 g, 95% confidence interval [CI] − 610 to − 270 g [18], to − 31 g, 95%CI − 57 to − 24 g [19], compared to sedentary controls) or LGA status after exposure to prenatal exercise pooled randomized trials and observational studies [18], included interventions that combined exercise and dietary changes [20], or used fixed effect models to analyze the data [19]. Fixed effect models assume that one true effect size is shared by all the included studies regardless of the population or type of exercise studied. Thus, utilizing a fixed effects model considers less variability in the primary studies, and is more likely to reach statistical significance with a large enough sample size [21]. Random effect models should, thus, be preferred when dealing with complex physiological conditions like the effect of different prenatal exercises undergone by different populations on offspring parameters. Other reviews [12, 22, 23] did not find any significant impact of prenatal exercise on birth weight, but were also limited by their specific scope [12, 23] or restricted search strategy [22].

To overcome these limitations and update past reviews [12, 18–20, 22, 23], we synthesized evidence from randomized controlled trials and prospective cohort studies separately to assess the impact of prenatal exercise on offspring cardiometabolic risk factors including weight, adiposity, and blood pressure at birth and in childhood while investigating the effects of maternal body mass index (BMI) and training variables. The clinical question guiding this review was: “Does maternal exercise training elicit short- and/or long-term cardiometabolic health benefits in offspring, compared to no exercise training?”

## Methods

### Study Design

Using a protocol designed a priori (PROSPERO #CRD42015029163), we conducted our systematic review using methodological approaches outlined in the *Cochrane Handbook for Systematic Reviews* [24] and reported according to the Preferred Reporting Items for Systematic Reviews and Meta-Analyses (PRISMA) criteria [25]. The search was targeted to identify studies to address the specific research question: Do regular aerobic and/or resistance exercises during pregnancy, compared to no exercise, reduce the risk of cardiometabolic disease in offspring? We defined *regular exercise* as voluntary movements done to improve or maintain fitness on a weekly basis for at least a month. The review team, composed of researchers in the fields of physiology, exercise, and developmental origins of health and disease, formulated the review question, reviewed the search strategies and review methods, and provided input throughout the review process.

### Literature Search Strategy

We searched Medline (Ovid), EMBASE (Ovid), CENTRAL (the Cochrane Library—Wiley), from inception to May 2016 for studies on prenatal pregnancy and cardiometabolic outcomes in the offspring. We added Scopus (Elsevier), CINAHL (EBSCO), and SPORTDiscus (EBSCO) to our target databases following comments from reviewers and reran the search through all six databases in May 2017. The Cochrane Highly Sensitive Search Strategy [26] was used as a model for searching; we designed search strategies specific to each database (see example on PROSPERO registration page). In order to identify ongoing or planned trials, we searched the World Health Organization’s International Clinical Trials Registry Platform (ClinicalTrials.gov). In addition to electronic searching, we hand-searched the bibliographies of relevant narrative and systematic reviews as well as those of included studies for additional citations. The search was rerun in December 2017 to include additional citations. Reference management was performed in EndNote™ (ver. 16, Thompson Reuters).

### Study Selection

We used a two-step process for study selection. First, all titles and abstracts of search results were screened independently in duplicate (by LG, NH, JLH, and CO) to determine if a study met the general inclusion criteria. The same reviewers independently examined the full texts of relevant citations. Disagreements were resolved by discussion between the reviewers or by third-party adjudication (LG or DSK), as needed. We included only randomized controlled trials or prospective cohort studies examining the impact of exercise undergone during pregnancy (from conception to delivery) on offspring cardiometabolic outcomes (see Table 1 for detailed inclusion and exclusion criteria). We hoped to reduce bias by selecting studies with the best experimental and observational designs; observational cohorts were included with the expectation that follow up data may be provided to inform long term offspring outcomes. No other restrictions, including language or publication status, were considered. The primary outcome measure was birth weight. Secondary outcomes included offspring fat and lean mass at birth, LGA status and weight, fat and lean mass, blood pressure, dyslipidemia, and dysglycemia at any time in childhood.

**Table 1:** Inclusion/exclusion criteria

	Inclusion	Exclusion
Study design	Randomized controlled trial OR prospective cohort study (including historical registry-based cohorts where measures were done prospectively)	Any other studies design, e.g., case-control, quasi-experimental, cross-sectional, cluster randomized, retrospective studies (including prospective studies where prenatal exercise was measured retrospectively)
Population	Pregnant women	Non-human populations
Intervention (for RCTs)	Aerobic and/or resistance (strength-) training exercises ≥ 4 weeks in duration	Intervention > 60% non-aerobic/resistance training (e.g., yoga, pilates, pelvic exercises); Studies of acute exercise, or training < 4 weeks in duration
Exposure (for cohorts)	Aerobic and/or resistance (strength-) training exercises ≥ 4 weeks in duration	Exposures not distinguishing physical activity from aerobic/resistance training; studies not documenting and/or linking prenatal exercise to our offspring outcomes, eg studies only reporting aggregate data for exercisers and non-exercisers
Comparator	No prenatal exercise	Unequal controls, e.g., controls do not receive the same diet information as exercisers, controls also undergo exercise training but to a different degree, etc.
Outcomes (offspring only)	At birth: • Weight (primary outcome) • Body composition (fat and/or lean mass) • Large-for-gestational age status At follow up (any time after birth): • Weight • Body composition (fat and/or lean mass) • Blood pressure • Blood glucose • Blood lipids	Studies not reporting any of these offspring outcomes Studies reporting these outcomes in a non-extractable format and authors do not agree to share the original data
Timing	Studies done at any point in time	

### Data Extraction and Quality Assessment

Three reviewers (LG, NH, and JLH) extracted data from included full texts using a standardized and piloted data extraction form. Extracted data included *funding sources*, *demographics* of the enrolled mothers and children (country, gestational age at randomization, maternal age at randomization, pre-pregnancy BMI, maternal smoking status at randomization; child age at latest follow-up), *details of the prenatal exercise* (type of exercise: aerobic or resistance training, frequency, intensity, timing during pregnancy; exercise measure: self-report or supervised), and predetermined offspring *outcomes* as described above and in Tables 1 and 2. When a trial reported results for more than one time period, results at birth and at the longest follow up were extracted separately. Intent-to-treat analysis was preferred when the data was presented accordingly. Data management was performed using Microsoft Word 2007 (Microsoft Corp).

**Table 2:** Included observational studies

Study	Country	Method	*N* (mother-child pairs)	Type of exercise	Intensity	Moment of exposure	Effect of prenatal exercise on offspring, compared to sedentary mothers^a^
Hatch 1993 [31]	USA	Two-centers cohort	200	Aerobic, Resistance	Vigorous	Throughout pregnancy	Adjusted birth weight difference: β = + 276 g (95% CI 54, 497)
Johnson 1994 [33]	USA	Multi-center cohort	234	Aerobic	Not specified	Not specified	Birth weight mean difference: + 109.2 g (*p* < 0.05)
Sternfeld 1995 [42]	USA	Institute-based, no GDM	139	Aerobic	Moderate to vigorous	Throughout pregnancy	Birth weight mean difference: + 68 g (*p* > 0.05)
Magann 2002 [37]	USA	Convenience sample	750	Aerobic	Moderate	Conception to 28 GW	Birth weight mean difference: -42 g (*p* = 0.87)
Nieuwen-huijsen 2002 [39]	England	Population-based	11,462	Aerobic	Not specified	18–20 weeks of pregnancy	Birth weight mean difference: + 16.7 g (95% CI − 11.4, 44.9)
Duncombe 2006 [29]	Australia	Convenience sample	148	Aerobic, Resistance	Vigorous (HR > 140 bpm)	GW 16 to 38	Birth weight mean difference: − 47 g (*p* = 0.49)
Snapp 2008 [41]	USA	Institute-based GDM cohort	75,160	Aerobic	Moderate	Throughout pregnancy	Birth weight: no difference LGA prevalence in exercisers: 0.73% (95% CI 0.10, 5.18%)
Juhl, AJOG, 2010 [35]	Denmark	Population-based (non-smokers only) registry study	58,435	Aerobic, resistance	NA	Throughout pregnancy	Adjusted birth weight mean difference: − 23 g (95% CI − 44 to − 1)
Juhl, Epidemiology 2010 [34]	Denmark	Population-based registry study	48,781	Aerobic	Light to vigorous	Not reported	Adjusted birth weight mean difference: − 7 (95% CI − 3 to 16)
Jukic 2010 [36]	USA	Convenience sample	1118	Aerobic, resistance	Vigorous	First trimester (conception to 12 GW)	Adjusted birth weight difference: β = + 40 g (95% CI − 154, 234)
Hegaard 2010 [32]	Denmark	One-center cohort	3961	Aerobic, resistance	Moderate to vigorous	At 16 and 30 GW	Adjusted birth weight mean difference: − 2 g (95% CI − 47, 42; 16 GW), and − 9 (95% CI − 62, 43; 30 GW)
Fleten 2010 [30]	Denmark	Population-based registry study	43,705	Aerobic, resistance	Light to moderate (self-report)	From conception to 17 GW; from 17 to 30 GW	Adjusted birth weight difference: β = − 0.72 g (95%CI − 1.3, − 0.1; 17 GW), and β = − 1.4 g (95% CI − 2.0, − 0.8; 17 to 30 GW)
Schou Andersen 2012 [40]	Denmark	Population-based registry study	40,280	Aerobic, resistance	Not specified	GW 16 and 36	Birth weight mean difference: − 15 g (*p* < 0.005) Unadjusted BMI difference at 7.1 years: − 0.1 kg/m^2^, *p* < 0.001
Millard 2013 [38]	England	Population-based	4665	Aerobic, resistance	Not specified	GW 18	Adjusted variables at 15.5 years: SBP: β = 0.02 (95% CI − 0.3, 0.3) LDLc: β = − 0.003 (95% CI − 0.022, 0.017) HDLc: β = − 0.001 (95% CI − 0.011, 0.009) FG: β = − 0.01 (95% CI − 0.03, 0.001) BMI: β = 0.02 (− 0.07, 0.12)
Bisson 2017 [28]	Canada	Population-based	1913	Aerobic, resistance	Not specified	First, second and third trimester	Adjusted birth weight difference: β = − 2.20 g (95% CI − 4.4, 0.01, 1st trimester) β = − 0.3 g (95% CI − 2.5, 2.0, 2nd trimester) β = 0.34 g (95% CI − 2.4, 3.1, 3rd trimester) LGA risk: RR 0.83 (95% CI 0.67, 1.02; 1st trimester)

*BMI* body mass index, *FG* fasting glucose, *GDM* gestational diabetes mellitus, *GW* gestational weeks, *HDLc* high-density lipoprotein cholesterol, *LGA* large-for-gestational-age, *LDLc* low-density lipoproteins cholesterol, *SBP* systolic blood pressure

^a^A positive difference means that the mean birth weight was higher in the exercise group than in the sedentary group

We evaluated the internal validity of included studies with the Cochrane Collaboration’s Risk of Bias tools [24, 27], in duplicate (LG and CO). Study authors were contacted if data as published was incomplete for the needs of the review.

### Data Synthesis and Analysis

We quantitatively analyzed data from the included studies using Review Manager (RevMan version 5.3.5, the Cochrane Collaboration). Pooled dichotomous data, calculated based on the generic inverse variance method, are presented as a risk ratio (RR) and pooled continuous data are expressed as a mean difference (MD), with 95% confidence intervals (CI). Only random effects models were used as populations and interventions varied. Statistical heterogeneity was explored and quantified using the *I*^2^ test. After validation against the associated protocols and/or trial registration information available, evidence of selective reporting found for any of the included trials was marked in the risk of bias assessment. A sensitivity analysis grouping only studies with low or unclear risk of bias was done for the primary outcome. Our pre-defined sub-group analyses, which were planned only for our primary outcome (birth weight) and only with data from randomized controlled trials (RCTs), were maternal pre-pregnancy activity level (inactive vs. active), maternal pre-pregnancy BMI, type of intervention (resistance training only vs. aerobic training only vs. combined), timing of exercise (starting in the first half of pregnancy, i.e., < 20 gestational weeks, vs. starting after the first half, i.e., ≥ 20 gestational weeks), country of origin, and internal validity (high vs. moderate/low quality). These sub-groups were intended to help pinpoint which components, if any, were responsible for the effect observed. Relevant components could then be highlighted in research and clinical interactions to promote better effectiveness. All tests of statistical inference reflect a two-sided α of 0.05.

## Results

### Search Results

**Fig. 1:**
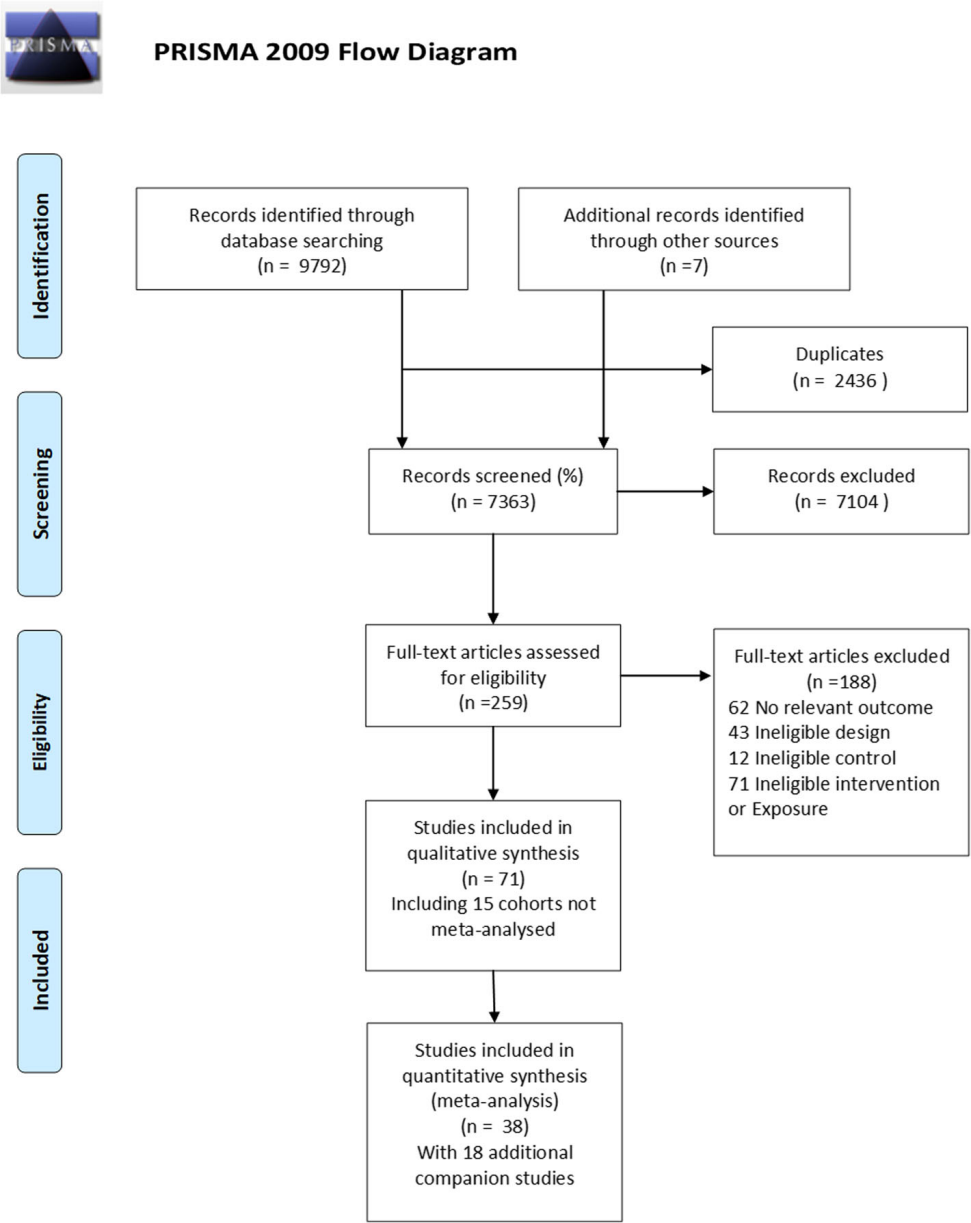
Flow diagram of literature search and study selection according to the 2009 Preferred Reporting Items of Systematic Reviews and Meta-Analyses (PRISMA) with modifications [25]. *Companion articles represent reports involving the same study population for the same intervention trial, including conference abstracts reporting findings from previous or subsequent full-length publications

Of 9804 citations identified through the literature search, 54 unique studies conducted between 1993 and 2017 were included, of which 15 were cohort studies [28–42] and 39 RCTs [43–81] with 19 companion publications [82–100] (see Fig. 1 for the flow chart and Tables 2 and 3 for studies characteristics). Nine additional studies not included in the previous meta-analyses were included [28, 51, 52, 55, 65, 70, 76, 77, 80]. Most studies (12) were conducted in the USA [31, 33, 36, 37, 41, 42, 47, 49, 61, 66, 73, 92]; others were from Australia and New Zealand [29, 62, 101], Brazil [43, 52, 53, 63, 68, 74, 81], Canada [28, 65], Colombia [72], Croatia [80], Denmark [30, 32, 34, 35, 40], Finland [54], Iran [56, 57], Kosovo [67], The Netherlands [69], Norway [55, 60, 102], Spain [44–46, 48, 50, 70, 79], Sweden [71], and the UK [38, 39, 76]. No study recruited previously active women, 16 recruited inactive women [43, 44, 46, 49, 52, 53, 55–58, 66–68, 70, 73, 92, 102], and the rest did not use previous exercise levels as a criterion. Most studies did not consider maternal BMI in their inclusion criteria, although eight specifically recruited women with overweight and/or obesity [51, 55, 64, 68, 69, 75, 77, 81], and three [49, 57, 65] women without overweight. The majority of experimental trials included healthy pregnant women, except five who targeted women with type 1 [61] or gestational diabetes [47, 52, 69, 74, 80], whereas observational cohorts were locally representative [31, 32, 41, 42], nationally representative [28, 30, 34, 35, 38–40], or convenience samples [29, 36, 37]. The funnel plot indicates that studies where exposure to prenatal exercise reduced birth weight were probably more likely to be published, at least for the smaller studies (Additional file 1: Fig. S1; especially seen for smaller studies). Concerning internal validity, most trials were adjudicated as of low or unclear risk of bias [43–46, 49, 52, 53, 57–59, 63, 67, 68, 74, 75, 83] (see Additional file 2: Fig. S2). The remaining trials were considered as high risk of bias due to their high dropout rates and unclear/inefficient blinding methods. All cohorts but one [29] were adjudicated at serious risk of bias, usually due to selection bias or missing data (Additional file 3: Fig. S3).

**Table 3:** Included randomized controlled trials

First author and year	Country	Population characteristics	Timing of intervention	Intervention type	Frequency	Intensity	Supervised	Sample size neonates (E/C)	Neonate outcome		
									Weight	FM	LGA
Erkkola 1975 [54]	Finland	Healthy	10–14 GW To 38 GW	Aerobic	3×/wk 60 min	Moderate to Vigorous ~140 bpm HR and fatigue	NR	44 (23/21)	**●**		
Hollings- worth 1987 [61]	USA	Type 1 diabetes	13 GW to 37–40 GW	Aerobic	Daily 20 min after each meal	NR	No	33 (13/21)	**●**		
Avery 1997 [47]	USA	GDM sedentary (exercise <2×/wk)	28 GW to delivery	Aerobic walking and cycling	3-4×/wk 30 min	Moderate < 70% HR max	Partial	29 (15/14)	●		
Kihlstrand 1999 [78]	Sweden	Healthy	20 GW to delivery	Aerobic	1×/wk, 30 min	NR	Yes	241 (122/119)	●		
Clapp 2000 [49]	USA	Healthy Did not regularly exercise (not defined)	8 GW to delivery	Aerobic	3-5×/wk 20 min	Moderate 55–60% VO_2_ max	Yes	46 (22/24)	●	●	
Marquez-Sterling 2000 [66]	USA	No regular exercise in the last year	18–20 GW to delivery	Aerobic	3×/wk 60 min	Moderate to vigorous 120–156 bpm	Yes	15 (9/6)	●		
Garshabi 2005 [56]	Iran	Healthy No history of exercise	17–22 GW to 29–34 GW	Aerobic and resistance	3×/wk 60 min	Moderate < 140 bpm	Yes	212 (107/105)	●		
Santos 2005 [81]	Brazil	Healthy 25 kg/m^2^ < BMI < 30 kg/m^2^	18 GW to NR	Aerobic and resistance	3×/wk 40–45 min	Moderate 50–60% HR max < 140 bpm	Yes	72 (37/35)	●		
Baciuk 2008 [43]	Brazil	No regular exercise	18–20 GW to delivery	Aerobic	3×/wk 50 min	Moderate < 70% HR max	Yes	70 (33/37)	●		
Barakat 2009 [44]	Spain	Healthy Sedentary (exercise < 20 min, 3×/wk)	12–13 GW to 38–39 GW	Resistance	3×/wk. 35–40 min	Light to vigorous < 60–< 80% HR max 10–12 Rep range	Yes	142 (72/70)	●		
De Barros 2010 [52]	Brazil	GDM Sedentary (IPAQ)	31 GW to delivery	Resistance	3×/wk 30–40 min	Moderate to vigorous 5–6 RPE/10	Partial	64 (32/32)	●		
Hopkins 2010 [62]	New Zealand	Healthy	2 GW to 36 GW or delivery	Aerobic	≤ 5 days 40 min	Moderate to vigorous 65% VO_2_ max	Partial	84 (47/37)	●	●	●
Haakstad 2011 [59]	Norway	Healthy No structured exercise (> 60 min 1×/wk)	17–18 GW 36–38 GW	Aerobic and resistance	2-7×/wk 60 min	Moderate to vigorous 12–14 RPE	Partial	105 (52/53)	●		
Nascimento 2011 [68]	Brazil	BMI ≥ 26 kg/m^2^	17 GW to delivery	Aerobic and resistance	6×/wk 40 min	Moderate < 140 bpm	Partial	82 (40/42)	●		●
De Oliveria Melo 2012 [53]	Brazil	Inactive Healthy	13 or 20 GW to delivery	Aerobic	3×/wk > 15 min	Moderate to vigorous 60–80% HR max Borg RPE 12–16	Yes	171 (54/60/57)	●		●
Oostdam 2012 [69]	Netherlands	BMI ≥ 25 kg/m^2^ with additional risk factor for GDM	15 GW to delivery	Aerobic and resistance	2×/wk 60 min	Moderate to vigorous 12–14 RPE	Yes	105 (52/53)	●		●
Pinzon 2012 [72]	Colombia	Healthy	16–20 GW to 32–36 GW	Resistance	3×/wk 60 min	Light to moderate 55–75% HR max	Yes	35 (18/17)	●		
Price 2012 [73]	USA	BMI < 39 kg/m^2^ No exercise (≥ 1x/wk for 6 months)	12–14 GW to 36 GW	Aerobic and resistance	4×/wk 45–60 min	Moderate to vigorous 12–14 RPE	Partial	62 (31/31)	●		
Barakat 2013 [46]	Spain	Healthy (exercise < 4×/wk)	6–9 GW to 38–39 GW	Aerobic, resistance	3×/wk 40–45 min	Light to moderate 60–75% HR max 10–12 Rep range	Yes	290 (138/152)	●		
Kasawara 2013 [63]	Brazil	Chronic HTN or history of preeclampsia	12–20 GW to delivery	Aerobic	1×/wk 30 min	Light to moderate 20% above resting HR to < 140 bpm	Yes	103 (53/50)			●
Ruiz 2013 [79]	Spain	Exercise < 20 min < 3×/wk	9 GW to 38–39 GW	Aerobic and resistance	3×/wk, 25–30 min	Light to moderate < 60% HRmax Borg RPE 10–12	Yes	962 (481/481)	●		
Barakat 2014 [48]	Spain	Healthy	9–13 GW to 39–40 GW	Aerobic, resistance	3×/wk 55–60 min	Moderate 55–60% HR max Borg RPE 12–13	Yes	200 (107/93)	●		
Cordero 2014 [50]	Spain	Healthy	10–14 GW to delivery	Aerobic and resistance	3×/wk, 32 min	Moderate to vigorous < 60% HR max Borg RPE 12–14	Yes	257 (101/156)	●		
Ghodsi 2014 [57]	Iran	BMI 19.8–26 kg/m^2^ No regular exercise	20–26 GW to 38 GW	Aerobic	3×/wk 15 min	Light to moderate 50–60% HR max	No	80 (40/40)	●		
Kong 2014 [64]	USA	BMI ≥ 25 kg/m^2^ (< 30 min, 3×/wk)	12–15 GW To 35 GW	Aerobic	5×/wk. 30 min	Moderate (step counts)	No	37 (18/19)	●		
Murtezani 2014 [67]	Kosovo	Healthy (exercise < 20 min, 3×/wk)	14–20 GW to delivery	Aerobic and resistance	3×/wk 40–45 min	Moderate to vigorous 12–14 RPE	Yes	63 (30/33)	●		
Petrov Fieril 2014 [71]	Sweden	Healthy	14 GW to 25 GW	Resistance	2×/wk 60 min	Moderate to vigorous (self-selected)	No	72 (38/34)	●		
Hellenes 2015 [60]	Norway	Healthy	18–22 GW to 32–36 GW	Aerobic and resistance	3×/wk 45–60 min	Moderate to vigorous 13–14 RPE	Partial	855 (429/426)	●		
Ramos 2015 [74]	Brazil	GDM	24–28 GW to 38 GW	Aerobic	3×/wk 50 min	NR	NR	6 (2/4)	●		
Ussher 2015 [76]	United Kingdom	Smokers (> 4 cigarettes/day prior to pregnancy)	10–24 GW for 8 GW	Aerobic	1-2×/wk 30 min	Moderate (self-report)	Yes	713 (354/359)	●		
Barakat 2016 [45]	Spain	Healthy	9–11 GW to 38–39 GW	Aerobic, resistance	3×/wk 50–55 min	Moderate to vigorous < 70% HR max Borg RPE 12–14	Yes	765 (382/383)	●		
Guelfi 2016 [58]	Australia	History of GDM in previous pregnancies with no structured exercise	14 to 28 GW	Aerobic	3×/wk 30-60 min	Moderate to vigorous intervals 65–85% HR max	Yes	172 (87/85)	●		●
Perales 2016 [70]	Spain	Pre gestational exercise < 4×/ wk. or current exercise ≤ 2×/wk 20 mins	9–11 GW To delivery	Aerobic, Resistance	3×/wk 55-60 min	Light to moderate 55–60% HR max	Yes	166 (83/83)	●		
Seneviratne 2016 [75]	New Zealand	BMI ≥ 25 kg/m^2^	20 GW to 35 GW	Aerobic	3 5×/wk 15–30 min	Moderate 40–59% VO_2_R	No	74 (37/37)	●		●
Daly 2017 [51]	Ireland	No diabetes BMI > 30 kg/m^2^	12 GW to 6 wk. post-partum	Aerobic and resistance	3×/wk 30–40 min	NR	Yes	87 (44/43)	**●**		
Labonte 2017 [65]	Canada	Healthy BMI 18.5–25 kg/m^2^	2nd to 3rd trimester	Aerobic	3×/wk 20 min	Moderate 55% VO_2_max	Partial	18 (19/8)	**●**		
Garnaes 2017 [55]	Norway	BMI > 28 kg/m^2^ (exercise < 2×/week)	12–18 GW to delivery	Aerobic and resistance	3–5/wk 60 min	Moderate to vigorous 12–15 RPE	Partial	74 (38/36)	●		
Sklempe 2017 [80]	Croatia	GDM	22–26 GW to delivery	Aerobic and resistance	2–7×/wk 40–45 min	Moderate 65–75% HR max Borg RPE 13–14	Partial	38 (18/20)	**●**		
Wang 2017 [77]	China	BMI > 24 kg/m^2^	8–12 GW to delivery	Aerobic	3×/wk 30–60 min	Light to vigorous intervals 50–85% HR max	Yes	226 (112/114)	**●**		**●**

Outcomes columns with dot indicate the specific outcome was reported by the study. *BMI* body mass index, *Bpm* beat per minute, *FM* fat mass, *GDM* gestational diabetes mellitus, *GW* gestational weeks, *HR* heart rate, *LGA* large-for-gestational age, *NR* not reported, *RPE* rating of perceived exertion, *VO*_2_*max* maximal oxygen consumption, *VO2R* oxygen uptake reserve, *wk* week

### Evidence from Observational Studies

#### Primary Outcome: Birth weight

Fifteen observational studies investigated the relationship between exercise in pregnancy and offspring cardiometabolic outcomes (see Table 2). They were too heterogenous in terms of exposure assessment and outcome reporting to be meta-analyzed. For the primary outcome, two cohorts found an increased average birth weight after exposure to exercise [31, 33] (from + 109 g, *p* < 0.05 to + 276 g, 95%CI 54, 497, *n* = 434), nine found no impact [28, 29, 32, 34, 36, 37, 39, 41, 42] (*n* = 143,432), and three lower average birth weight after prenatal exercise [30, 35, 40] (from − 23 g, 95% CI − 44 to − 1, to − 0.72 g, 95% CI − 1.3 to − 0.1 g, *n* = 142,420).

#### Secondary Outcomes

Two studies found investigated the risk of being born LGA after exposure to prenatal exercise. Although both found a similar reduction in risk, none were statistically significant (RR 0.83, 95% CI 0.67, 1.02, *n* = 1913 [28]; prevalence 0.73%, 95% CI 0.10, 5.18%; *n* = 20,458 [41]). Two studies reported long-term secondary outcomes after following up offspring at 7.1 [40] and 15.5 years old [38]. No significant relationships were found with exposure to prenatal exercise on BMI, blood pressure, blood lipids, or fasting glucose in these studies after adjustment for confounders.

### Evidence from Randomized Controlled Trials

#### Primary Outcome

**Fig. 2:**
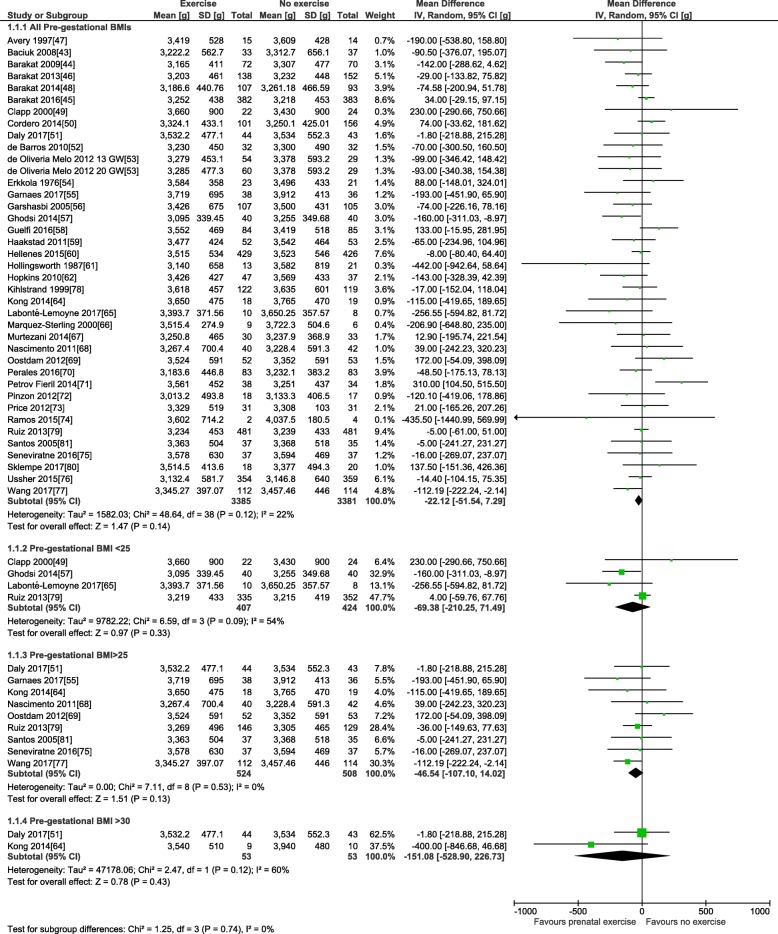
Forest plot of pooled mean differences for birth weight after exposure to prenatal exercise vs. no exercise

**Fig. 3:**
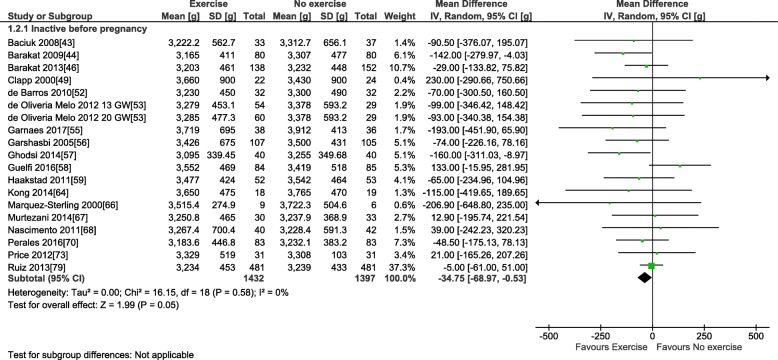
Forest plot of pooled mean differences for birth weight after exposure to prenatal exercise vs. no exercise; sub-grouping by activity level before pregnancy: active vs. inactive

**Fig. 4:**
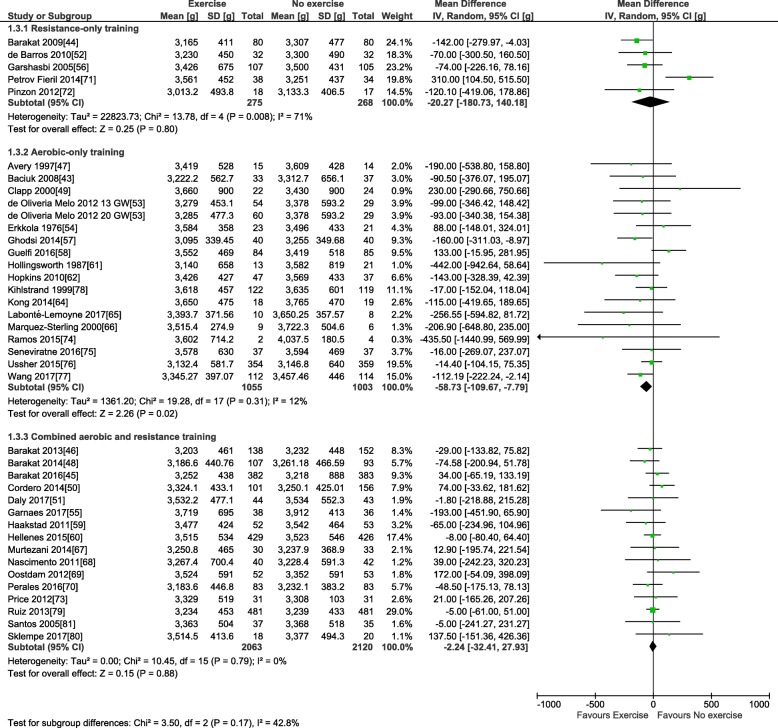
Forest plot of pooled mean differences for birth weight after exposure to prenatal exercise vs. no exercise; sub-grouping by type of intervention: resistance training only, aerobic training only, or combined resistance and aerobic training

**Fig. 5:**
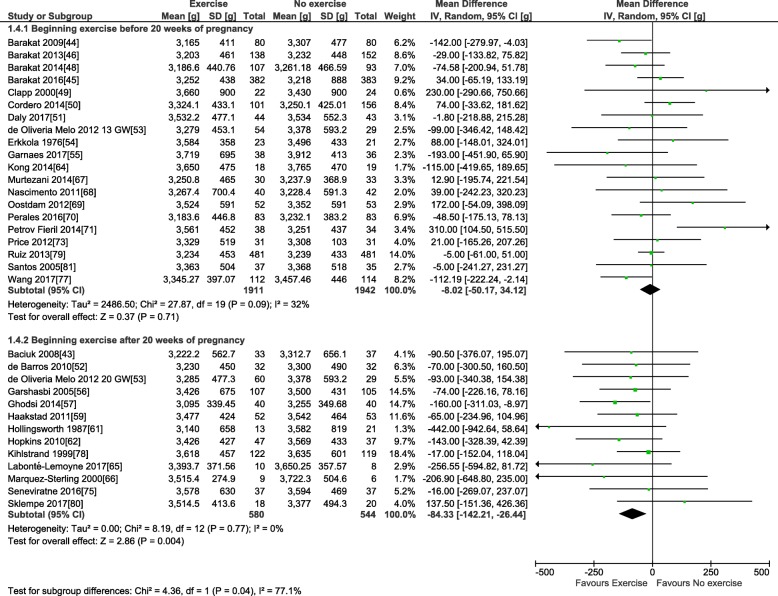
Forest plot of pooled mean differences for birth weight after exposure to prenatal exercise vs. no exercise; sub-grouping by timing of intervention: first half of pregnancy (< 20 gestational weeks) vs. second half of pregnancy (≥ 20 gestational weeks)

**Fig. 6:**
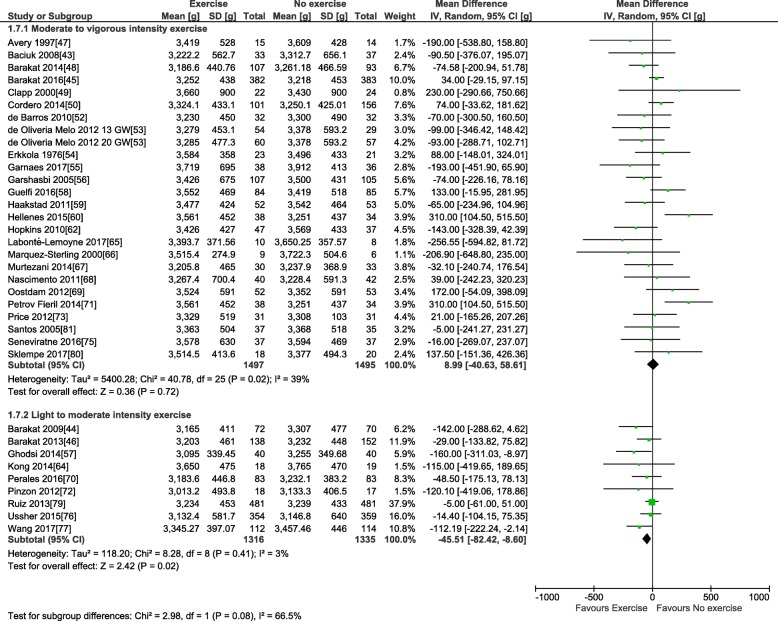
Forest plot of pooled mean differences for birthweight after exposure to prenatal exercise vs. no exercise; sub-grouping by intensity level : Moderate to vigorous and Light to moderate

**Fig. 7:**
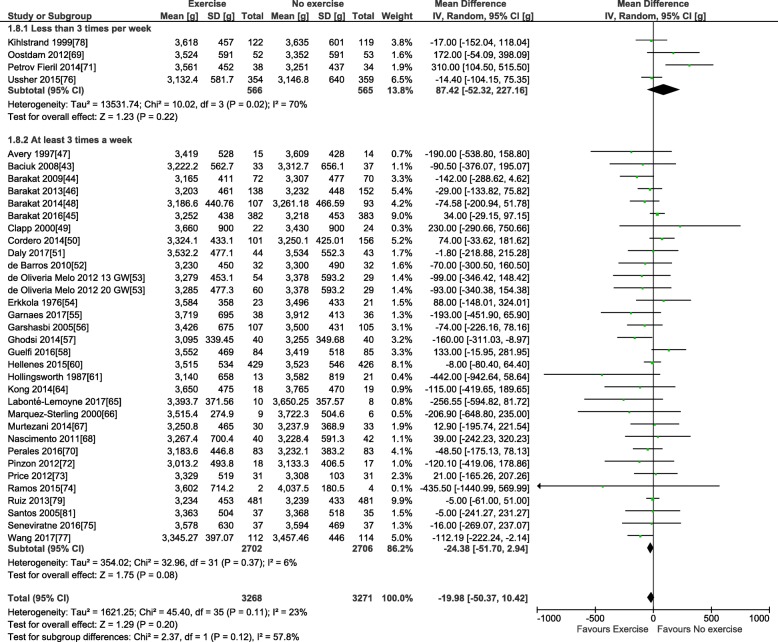
Forest plot of pooled mean differences for birthweight after exposure to prenatal exercise vs. no exercise; sub-grouping by frequency of the exercise: Less than 3 times per week, and At least 3 times per week

For the primary outcome, 38 trials involving 6766 pregnant women provided data for the meta-analysis of birth weight (see Table 3). Compared to control condition (no prenatal exercise), there was no difference in birth weight after exercise interventions delivered in pregnancy at any time period, frequency, or intensity of exercise (mean difference (MD): − 22.1 g, 95% confidence interval [CI] − 51.5 to 7.3 g; *I*^2^ 22%; see Fig. 2). Restricting to the studies with healthy populations did not yield very different results (MD − 23.6 g, 95% CI − 54.7, 7.5; *I*^2^ 23%, 31 trials, *n* = 5777). The moderate statistical heterogeneity led us to conduct our pre-defined sub-group analyses. Sub-grouping by maternal BMI indicated that prenatal exercise had no effect according to pre-pregnancy BMI categories (see Fig. 2), either < 25 kg/m^2^ (MD − 69.4, 95% CI − 210.3, 71.5; *I*^2^ 54%; four trials, *n* = 831), > 25 kg/m^2^ (MD − 49.5, 95% CI − 112.1, 13.2; *I*^2^ 0%; eight trials, *n* = 960), or > 30 kg/m^2^ (MD − 151.1, 95% CI − 528.9, 226.7; *I*^2^ 60%; two trials; *n* = 106). Sub-grouping according to maternal pre-pregnancy activity level (inactive vs. active) indicated that prenatal exercise could reduce birth weight in previously inactive women (MD − 34.8 g, 95% CI − 69.0, − 0.5 g; *I*^2^ 0%; 18 trials, *n* = 2829); however, as no study specifically included active women, we could not assess that sub-group (Fig. 3). Sub-grouping according to type of exercise showed that aerobic-only training similarly reduced birth weight (MD − 58.7 g, 95% CI − 109.7, − 7.8; *I*^2^ 12%; 17 studies, *n* = 2058), but resistance training only (5 trials, *n* = 543), or combined regimens (16 trials, *n* = 4183) had non-significant effects (see Fig. 4). Prenatal exercise regimens starting after the 20th week of pregnancy marginally reduced birth weight (MD − 84.3 g, 95% CI − 142.2, − 26.4 g; *I*^2^ 0%, *n* = 1124), whereas interventions starting before this time had no impact on birth weight (20 studies, *n* = 3853, see Fig. 5). Interventions that were light to moderate intensity reduced birth weight (MD − 45.5 g, 95% CI − 82.4, − 8.6 g; *I*^2^ 3%; 9 trials, *n* = 2651) but not those that were moderate to vigorous intensity (25 trials, *n* = 2992; Fig. 6). Finally, frequency of exercise did not impact birth weight, whether interventions were less than three times a week (4 trials, *n* = 1131) or at least that frequent (31 trials, *n* = 5408; Fig. 7). Restricting to studies with low to moderate risk of bias did not yield different results (MD − 10.9, 95% CI − 42.1, 20.4; *I*^2^ 0%; 17 trials, *n* = 3418; data not shown); however, studies from developing countries were more likely to find that prenatal exercise reduced birth weight (MD − 78.7 g, 95% CI − 135.4, − 22.0 g; *I*^2^ 0%; 12 trials, *n* = 1120) compared to studies in developed countries (MD − 8.3 g, 95% CI − 43.8, 27.11 g; *I*^2^ 32%; 26 trials, *n* = 5646; data not shown).

#### Secondary Outcomes

**Fig. 8:**
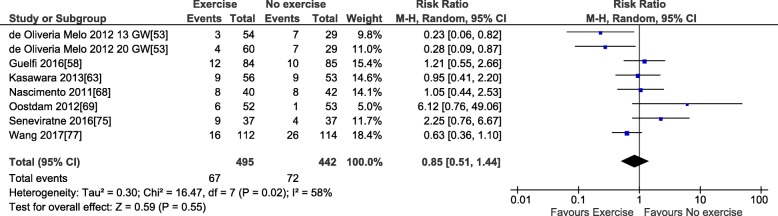
Forest plot of pooled risk ratios for large-for-gestational-age (LGA) status

**Fig. 9:**
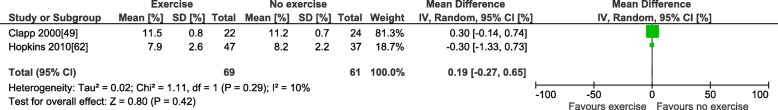
Forest plot of pooled mean differences for fat mass percentage after exposure to prenatal exercise vs. vno exercise

Only LGA status and fat mass at birth could be meta-analyzed. Data concerning the other outcomes were either not reported by more than one study or were not clinically homogenous enough to be pooled (e.g., collected at different ages). Prenatal exercise did not reduce the risk of LGA (RR 0.85, 95% CI 0.51, 1.44; *I*^2^ 58%; seven studies; *n* = 937; Fig. 8) nor impact fat mass percentage (MD 0.19, 95% CI − 0.27, 0.65%; *I*^2^ 10%; two studies; *n* = 130; Fig. 9). Qualitatively, the two studies that followed up offspring after birth did not indicate any significant impact of prenatal exercise on weight [62, 92] or fat mass [62, 92] at 17 days or 6 months old. No RCT reported on offspring blood pressure, blood glucose, or blood lipids.

## Discussion

Exercise is an established cornerstone for optimizing women’s metabolic health, and prenatal exercise is safe for mothers and fetus [103]. As evidence is accumulating that in utero exposures have a major influence on the fetus’ future cardiometabolic health [10], the positive maternal impacts of exercise on women’s cardiometabolic health have recently been posited to extend to exposed fetuses [23]. Although recent meta-analyses stated prenatal exercise might prevent giving birth to larger babies [18, 19, 22], we found contrasting results from both high quality observational cohorts and RCTs which indicated that prenatal exercise does not impact average birth weight in a significant manner. None of the included cohort studies found a clinically relevant birth weight difference (i.e., ≥ 300 g [104]) after exposure to various kinds of prenatal exercise. Thus, even though the methodological differences made it difficult to compare the studies and explain their opposing results (prenatal exercise increasing vs. decreasing birth weight), none of the reviewed cohorts reported clinically relevant impacts of prenatal exercise on birth weight. As few cohorts followed children into childhood or measured other variables than weight, the long-term impact of maternal exercise on offspring cardiometabolic health remains unclear. Long-term follow-up of pregnancy cohorts is needed to discern the influence of exercise in pregnancy and child health.

Similar to results observed by prospective cohorts, we did not find a clinically relevant effect of prenatal aerobic and/or strength training interventions on child birth weight, LGA status, or birth fat mass. Although prenatal exercise led to statistically significant birth weight reduction in some sub-group analyses, the mean effect varied from − 0.5 to − 84 g, which are clinically negligible impacts [104]. We were limited in our ability to examine the impact of prenatal exercise on other important health outcomes (childhood blood pressure, glucose, lipids, and fat mass) because they were not measured or reported by the trials, therefore the long-term impact of exposure to prenatal exercise on cardiometabolic health of offspring could not be assessed.

Birth weight is a very common marker of infant health due to its ease of measurement and its historically frequent association with future health outcomes [105]. Nonetheless, recent work in the field of developmental origins of health and disease indicate that weight is only a crude marker of health. For example, some studies indicate that offspring born small for gestational age are leaner later in life [106, 107] while others found that these offspring were at increased risk of obesity [108–110]. Likewise, some studies found increased markers of cardiometabolic risk in LGA offspring [111, 112] while others did not find evidence of increased risk [113–115]. Thus, in order to adequately assess the potentially protective effects of prenatal exercise on offspring cardiometabolic health, it is imperative to measure other relevant markers (e.g., body composition, blood glucose, and lipids) at birth and later in childhood. Our unexpected null results provide cautionary evidence that exercise by itself is not sufficient to impact birth weight, as some have argued [28, 116]. On the other hand, they suggest that women can safely participate in the type of activity they prefer (aerobic or resistance) at the intensity and frequency that suits them, which might increase adherence.

### Strengths and Limitations

Strengths of this systematic review include isolating the causal impact of prenatal exercise (vs. other interventions like dietary modifications); restricting to high-quality designs to reduce bias (prospective cohorts and RCTs); considering outcomes other than weight to assess the impact of prenatal exercise on offspring health; considering maternal and training variables not assessed by previous reviews (timing, intensity and frequency of intervention, maternal BMI, country of origin); using random effect models for all analyses; and using a protocol established a priori. Despite these strengths, the review has some limitations. First, only studies assessing aerobic and/or strength training were included, discarding studies where other forms of prenatal exercise (e.g., yoga [117]) were measured. This choice was made because current recommendations [118, 119] are focused on those two types of exercise. However, as > 80% of active pregnant women report engaging in some kind of aerobic training [120], we are confident our results are representative of real-life prenatal exercise habits and are therefore relevant for clinicians and researchers. Second, our predefined sub-groups addressed only one variable at a time (e.g., maternal BMI, timing of exercise). It is possible that evaluating the interaction by grouping according to many parameters (e.g., among women with a BMI > 25, those who starting exercising < 20 gestational weeks) through a meta-regression might be more informative. However, such analyses were not planned a priori so another study would be needed to answer this limitation. Third, there was marked heterogeneity in research designs, assessments of exercise dose (frequency, intensity, duration, adherence), and reports of offspring outcomes, making direct comparisons between studies difficult. Accordingly, we refrained from pooling results that we considered too heterogeneous and were careful in not over-interpreting the results.

### Evidence Gaps

It is imperative that future trials report determinants of offspring cardiometabolic health other than birth weight, such as adiposity, plasma glucose and lipids, and blood pressure early in life and ideally at multiple times throughout childhood to define the long term impact of exposure to prenatal exercise on offspring cardiometabolic health. Indeed, there are indications that higher blood pressure [121], glycemia [5], and dyslipidemia [122] early in life are related to future metabolic syndrome, diabetes, and cardiovascular diseases, whereas birth weight is a crude marker [113, 123]. Follow up of data for these parameters in childhood would provide important tools to public health authorities to help determine if and how prenatal exercise improves offspring cardiovascular risk factors in both the short and long term. Indeed, assessing offspring fat and lean mass might be more informative than only weight. Additionally, offspring should be periodically reassessed as there is a dearth of longitudinal data concerning offspring exposed to exercise interventions in the literature. A sample size calculation based on the RCTs included indicate that matched groups of at least 268 participants (536 participants total) are needed to detect a birth weight difference between groups at 90% power and with a 0.05 double-sided α. However based on our analyses, future interventions should include components other than exercise (such as a dietary intervention) if the intent is to have an impact on birth weight. Finally, more diverse participants in terms of pre-pregnancy activity level and body composition are needed in future studies to understand how exercise interventions in pregnancy modulate the relationship between maternal physiology, offspring body composition, and cardiometabolic health. Clinicians looking to counsel their clients might want to highlight that while prenatal exercise is perfectly safe for the baby, the best evidence currently available indicates it is not sufficient by itself to protect the child against cardiometabolic diseases.

## Conclusion

In summary, high-quality studies analyzed with conservative statistics show that the impact of prenatal exercise on birth weight is not clinically relevant. This impact might be more important in previously less active women and when the exercise program has light to moderate intensity and starts in the second half of pregnancy. Due to the scarcity of studies collecting parameters other than birth weight and/or following up offspring in childhood, there is limited evidence about the relationship of prenatal exercise and long-term offspring cardiometabolic health. Thus, there is a great need for the collection of data other than weight and for the long-term follow up of offspring exposed to exercise to better define the impact of prenatal exercise on offspring cardiometabolic risk throughout life. Researchers and clinicians intending to impact the health of the future generations should consider adding other components (such as dietary components) to their exercise interventions.

## Additional Files

Additional file 1: **Figure S1.** Funnel plot of included randomized controlled trials that contributed birth weight data, with each trial represented by a gray circle (*n* = 34). The horizontal axis represents the standardized mean difference. The vertical axis represents the standard error of the mean. Individual study results are represented by the open circles. The vertical line in the plot represents the pooled effect size. The poor symmetry specifically in smaller studies might indicate a publication bias favoring studies that found a reduction in birth weight following prenatal exercise. (EPS 77 kb)

Additional file 2: **Figure S2.** Summary of risk of bias for individual studies following the Cochrane tool. Low risk of bias is indicated by the plus sign, high risk of bias by the minus sign and unclear risk of bias by the question mark. (EPS 980 kb)

Additional file 3: **Figure S3.** Summary of risk of bias for individual studies following the Risk Of Bias In Non-randomized Studies of Interventions tool. The possible categories of risk of bias are: Low (green), Moderate (Mod; blue), Serious (red), Critical (gray), and No information (NI; yellow). (TIF 111 kb)

## Abbreviations

BMI: Body mass index
CI: Confidence interval
LGA: Large for gestational age
MD: Mean difference
RCT: Randomized controlled trials
RR: Risk ratio

## References

[1] Rodd C, Sharma AK. Recent trends in the prevalence of overweight and obesity among Canadian children. Can Med Assoc J. 2016; 10.1503/cmaj.150854. 10.1503/cmaj.150854 PMC5026530 27160875

[2] Ogden CL, Carroll MD, Kit BK, Flegal KM (2014). Prevalence of childhood and adult obesity in the United States, 2011-2012. JAMA.

[3] Kim J, Lee I, Lim S (2017). Overweight or obesity in children aged 0 to 6 and the risk of adult metabolic syndrome: a systematic review and meta-analysis. J Clin Nurs.

[4] Hales CN, Barker DJ (2001). The thrifty phenotype hypothesis. Br Med Bull.

[5] Boney CM, Verma A, Tucker R, Vohr BR (2005). Metabolic syndrome in childhood: association with birth weight, maternal obesity, and gestational diabetes mellitus. Pediatrics.

[6] Huang R-C, de Klerk NH, Smith A, Kendall GE, Landau LI, Mori TA (2011). Lifecourse childhood adiposity trajectories associated with adolescent insulin resistance. Diabetes Care.

[7] Silverman BL, Rizzo TA, Cho NH, Metzger BE (1998). Long-term effects of the intrauterine environment. The Northwestern University diabetes in pregnancy center. Diabetes Care.

[8] Barker DJ, Gluckman PD, Godfrey KM, Harding JE, Owens JA, Robinson JS (1993). Fetal nutrition and cardiovascular disease in adult life. Lancet.

[9] Nicholas LM, Rattanatray L, Morrison JL, Kleemann DO, Walker SK, Zhang S (2014). Maternal obesity or weight loss around conception impacts hepatic fatty acid metabolism in the offspring. Obesity (Silver Spring).

[10] Winett L, Wallack L, Richardson D, Boone-Heinonen J, Messer L (2016). A framework to address challenges in communicating the developmental origins of health and disease. Curr Environ Health Rep.

[11] Blaize AN, Pearson KJ, Newcomer SC (2015). Impact of maternal exercise during pregnancy on offspring chronic disease susceptibility. Exercise & Sport Sciences Reviews.

[12] Kramer MS, McDonald SW (2006). Aerobic exercise for women during pregnancy. Cochrane database of systematic reviews (Online).

[13] Loprinzi PD, Fitzgerald EM, Woekel E, Cardinal BJ (2013). Association of physical activity and sedentary behavior with biological markers among U.S. pregnant women. J Women's Health (Larchmt).

[14] Mudd LM, Evenson KR (2015). Review of impacts of physical activity on maternal metabolic health during pregnancy. Curr Diab Rep.

[15] Barakat R, Cordero Y, Coteron J, Luaces M, Montejo R (2012). Exercise during pregnancy improves maternal glucose screen at 24-28 weeks: a randomised controlled trial. Br J Sports Med.

[16] Callaway LK, Colditz PB, Byrne NM, Lingwood BE, Rowlands IJ, Foxcroft K (2010). Prevention of gestational diabetes: feasibility issues for an exercise intervention in obese pregnant women. Diabetes Care.

[17] Ong MJ, Guelfi KJ, Hunter T, Wallman KE, Fournier PA, Newnham JP (2009). Supervised home-based exercise may attenuate the decline of glucose tolerance in obese pregnant women. Diabetes Metab.

[18] Leet T, Flick L (2003). Effect of exercise on birthweight. Clin Obstet Gynecol.

[19] Wiebe HW, Boule NG, Chari R, Davenport MH (2014). The impact of prenatal exercise on fetal growth: a meta-analysis. Can J Diabetes.

[20] Thangaratinam S, Rogozinska E, Jolly K, Glinkowski S, Roseboom T, Tomlinson JW (2012). Effects of interventions in pregnancy on maternal weight and obstetric outcomes: meta-analysis of randomised evidence. BMJ.

[21] Mansourian M, Mohammadi R, Marateb HR, Yazdani A, Goodarzi-Khoigani M, Molavi S (2017). Comprehensive maternal characteristics associated with birth weight: Bayesian modeling in a prospective cohort study from Iran. Journal of Research in Medical Sciences.

[22] Perales M, Santos-Lozano A, Ruiz JR, Lucia A, Barakat R (2016). Benefits of aerobic or resistance training during pregnancy on maternal health and perinatal outcomes: a systematic review. Early Hum Dev.

[23] Moyer C, Reoyo OR, May L (2016). The influence of prenatal exercise on offspring health: a review. Clinical Medicine Insights Women's health.

[24] Higgins JPT, Green S, Cochrane C (2008). Cochrane handbook for systematic reviews of interventions.

[25] Moher D, Liberati A, Tetzlaff J, Altman DG (2010). Preferred reporting items for systematic reviews and meta-analyses: the PRISMA statement. International journal of surgery (London, England).

[26] Robinson K, Dickersin K (2002). Development of a highly sensitive search strategy for the retrieval of reports of controlled trials using PubMed. Int J Epidemiol.

[27] Sterne JA, Hernán MA, Reeves BC, Savović J, Berkman ND, Viswanathan M, et al. ROBINS-I: a tool for assessing risk of bias in non-randomised studies of interventions. BMJ. 2016;355 10.1136/bmj.i4919. 10.1136/bmj.i4919 PMC5062054 27733354

[28] Bisson M, Croteau J, Guinhouya BC, Bujold E, Audibert F, Fraser WD (2017). Physical activity during pregnancy and infant's birth weight: results from the 3D birth cohort. BMJ Open Sport Exerc Med.

[29] Duncombe D, Skouteris H, Wertheim EH, Kelly L, Fraser V, Paxton SJ (2006). Vigorous exercise and birth outcomes in a sample of recreational exercisers: a prospective study across pregnancy. Aust N Z J Obstet Gynaecol.

[30] Fleten C, Stigum H, Magnus P, Nystad W (2010). Exercise during pregnancy, maternal prepregnancy body mass index, and birth weight. Obstet Gynecol.

[31] Hatch MC, Shu XO, McLean DE, Levin B, Begg M, Reuss L (1993). Maternal exercise during pregnancy, physical fitness, and fetal growth. Am J Epidemiol.

[32] Hegaard HK, Petersson K, Hedegaard M, Ottesen B, Dykes AK, Henriksen TB (2010). Sports and leisure-time physical activity in pregnancy and birth weight: a population-based study. Scand J Med Sci Sports.

[33] Johnson AA, Knight EM, Edwards CH, Oyemade UJ, Cole OJ, Westney OE (1994). Selected lifestyle practices in urban African American women—relationships to pregnancy outcome, dietary intakes and anthropometric measurements. J Nutr.

[34] Juhl M, Kogevinas M, Andersen PK, Andersen AM, Olsen J (2010). Is swimming during pregnancy a safe exercise?. Epidemiology.

[35] Juhl M, Olsen J, Andersen PK, Nohr EA, Andersen AM (2010). Physical exercise during pregnancy and fetal growth measures: a study within the Danish National Birth Cohort. American Journal of Obstetrics & Gynecology..

[36] Jukic A, Evenson K, Daniels J, Herring AH, Wilcox A, Hartmann K (2010). A prospective study of the association between vigorous physical activity during pregnancy and length of gestation and birthweight. Am J Epidemiol.

[37] Magann EF, Evans SF, Weitz B, Newnham J (2002). Antepartum, intrapartum, and neonatal significance of exercise on healthy low-risk pregnant working women. Obstet Gynecol.

[38] Millard LAC, Lawlor DA, Fraser A, Howe LD (2013). Physical activity during pregnancy and offspring cardiovascular risk factors: findings from a prospective cohort study. BMJ Open.

[39] Nieuwenhuijsen MJ, Northstone K, Golding J (2002). Swimming and birth weight. Epidemiology (Cambridge, Mass).

[40] Schou Andersen C, Juhl M, Gamborg M, Sorensen TI, Nohr EA (2012). Maternal recreational exercise during pregnancy in relation to Children's BMI at 7 years of age. International Journal of Pediatrics.

[41] Snapp CA, Donaldson SK (2008). Gestational diabetes mellitus: physical exercise and health outcomes. Biological Research for Nursing.

[42] Sternfeld B, Quesenberry CP, Eskenazi B, Newman LA (1995). Exercise during pregnancy and pregnancy outcome. / Pratique de l ' exercice physique pendant la grossesse et son incidence sur la grossesse. Medicine & Science in Sports & Exercise.

[43] Baciuk EP, Pereira RI, Cecatti JG, Braga AF, Cavalcante SR. Water aerobics in pregnancy: cardiovascular response, labor and neonatal outcomes. Reprod Health. 2008; 10.1186/1742-4755-5-10. 10.1186/1742-4755-5-10 PMC2613131 19025579

[44] Barakat R, Lucia A, Ruiz JR. Resistance exercise training during pregnancy and newborn's birth size: a randomised controlled trial. International journal of obesity. 2005) 2009; 10.1038/ijo.2009.150. 10.1038/ijo.2009.150 19636320

[45] Barakat R, Pelaez M, Cordero Y, Perales M, Lopez C, Coteron J, et al. Exercise during pregnancy protects against hypertension and macrosomia: randomized clinical trial. Am J Obstet Gynecol. 2016; 10.1016/j.ajog.2015.11.039. 10.1016/j.ajog.2015.11.039 26704894

[46] Barakat R, Pelaez M, Lopez C, Montejo R, Coteron J. Exercise during pregnancy reduces the rate of cesarean and instrumental deliveries: results of a randomized controlled trial. Journal of maternal-fetal & neonatal medicine. 2013; 10.3109/14767058.2012.696165. 10.3109/14767058.2012.696165 22715981

[47] Avery MD, Leon AS, Kopher RA. Effects of a partially home-based exercise program for women with gestational diabetes. Obstet Gynecol. 1997;89(1):10-5. 10.1016/s0029-7844(97)84256-1 8990428

[48] Barakat R, Perales M, Bacchi M, Coteron J, Refoyo I (2014). A program of exercise throughout pregnancy. Is it safe to mother and newborn?. American journal of health promotion: AJHP.

[49] Clapp JF, Kim H, Burciu B, Lopez B. Beginning regular exercise in early pregnancy: effect on fetoplacental growth. Am J Obstet Gynecol. 2000; 10.1067/mob.2000.107096. 10.1067/mob.2000.107096 11120515

[50] Cordero FY, Mottola FM, Vargas FJ, Blanco FM, Barakat FR (2015). Exercise is associated with a reduction in gestational diabetes mellitus. Med Sci Sports Exerc.

[51] Daly N, Farren M, McKeating A, O'Higgins A, Mullaney L, Turner MJ (2017). Effect of a randomized controlled trial of an intensive medically supervised exercise program designed to improve maternal glucose control on gestational weight gain. Am J Obstet Gynecol.

[52] de Barros MC, Lopes MA, Francisco RP, Sapienza AD, Zugaib M (2010). Resistance exercise and glycemic control in women with gestational diabetes mellitus. Am J Obstet Gynecol.

[53] De Oliveria Melo AS, Silva JLP, Tavares JS, Barros VO, Leite DFB, Amorim MMR (2012). Effect of a physical exercise program during pregnancy on uteroplacental and fetal blood flow and fetal growth: a randomized controlled trial. Obstet Gynecol.

[54] Erkkola R, Mäkelä M (1976). Heart volume and physical fitness of parturients. Ann Clin Res.

[55] Garnaes KK, Nyrnes SA, Salvesen KA, Salvesen O, Morkved S, Moholdt T (2017). Effect of supervised exercise training during pregnancy on neonatal and maternal outcomes among overweight and obese women. Secondary analyses of the ETIP trial: a randomised controlled trial. PLoS One.

[56] Garshasbi A, Faghih Zadeh S (2005). The effect of exercise on the intensity of low back pain in pregnant women. Int J Gynecol Obstet.

[57] Ghodsi Z, Asltoghiri M (2014). Effects of aerobic exercise training on maternal and neonatal outcome: a randomized controlled trial on pregnant women in Iran. JPMA The Journal of the Pakistan Medical Association.

[58] Guelfi KJ, Ong MJ, Crisp NA, Fournier PA, Wallman KE, Grove JR (2016). Regular exercise to prevent the recurrence of gestational diabetes mellitus: a randomized controlled trial. Obstet Gynecol.

[59] Haakstad LA, Bo K (2011). Exercise in pregnant women and birth weight: a randomized controlled trial. BMC Pregnancy & Childbirth.

[60] Hellenes OM, Vik T, Løhaugen GC, Salvesen K, Stafne SN, Mørkved S, et al. Regular moderate exercise during pregnancy does not have an adverse effect on the neurodevelopment of the child. Acta paediatrica (Oslo, Norway : 1992). 2015; 10.1111/apa.12890. 10.1111/apa.12890 25471255

[61] Hollingsworth DR, Moore TR (1987). Postprandial walking exercise in pregnant insulin-dependent (type I) diabetic women: reduction of plasma lipid levels but absence of a significant effect on glycemic control. American Journal of Obstetrics & Gynecology.

[62] Hopkins SA, Baldi JC, Cutfield WS, McCowan L, Hofman PL. Exercise training in pregnancy reduces offspring size without changes in maternal insulin sensitivity. J Clin Endocrinol Metab. 2010; 10.1210/jc.2009-2255. 10.1210/jc.2009-2255 20335449

[63] Kasawara KT, Burgos CS, do Nascimento SL, Ferreira NO, Surita FG, Pinto ESJL (2013). Maternal and perinatal outcomes of exercise in pregnant women with chronic hypertension and/or previous preeclampsia: a randomized controlled trial. ISRN obstetrics and gynecology.

[64] Kong KL, Campbell CG, Foster RC, Peterson AD, Lanningham-Foster L (2014). A pilot walking program promotes moderate-intensity physical activity during pregnancy. Med Sci Sports Exerc.

[65] Labonte-Lemoyne E, Curnier D, Ellemberg D (2017). Exercise during pregnancy enhances cerebral maturation in the newborn: a randomized controlled trial. Journal of Clinical & Experimental Neuropsychology: official. J Int Neuropsychol Soc.

[66] Marquez-Sterling S, Perry AC, Kaplan TA, Halberstein RA, Signorile JF. Physical and psychological changes with vigorous exercise in sedentary primigravidae. Med Sci Sports Exerc. 2000; 10.1097/00005768-200001000-00010 10647530

[67] Murtezani A, Paçarada M, Ibraimi Z, Nevzati A, Abazi N (2014). The impact of exercise during pregnancy on neonatal outcomes: a randomized controlled trial. J Sports Med Phys Fitness.

[68] Nascimento SL, Surita FG, Parpinelli M, Siani S, Pinto e Silva JL (2011). The effect of an antenatal physical exercise programme on maternal/perinatal outcomes and quality of life in overweight and obese pregnant women: a randomised clinical trial. BJOG: An International Journal of Obstetrics and Gynaecology.

[69] Oostdam N, Poppel MN, Wouters MG, Eekhoff EM, Bekedam DJ, Kuchenbecker WK, et al. No effect of the FitFor2 exercise programme on blood glucose, insulin sensitivity, and birthweight in pregnant women who were overweight and at risk for gestational diabetes: results of a randomised controlled trial. BJOG : an international journal of obstetrics and gynaecology. 2012; 10.1111/j.1471-0528.2012.03366.x. 10.1111/j.1471-0528.2012.03366.x 22616913

[70] Perales M, Calabria I, Lopez C, Franco E, Coteron J, Barakat R. Regular exercise throughout pregnancy is associated with a shorter first stage of labor. Am J Health Promot. 2017; 10.4278/ajhp.140221-QUAN-79. 10.4278/ajhp.140221-QUAN-79 25615706

[71] Petrov Fieril K, Glantz A, Fagevik Olsen M (2015). The efficacy of moderate-to-vigorous resistance exercise during pregnancy: a randomized controlled trial. Acta Obstet Gynecol Scand.

[72] Pinzon DC, Zamora K, Martinez JH, Florez-Lopez ME, de Plata AC, Mosquera M (2012). Type of delivery and gestational age is not affected by pregnant Latin-American women engaging in vigorous exercise: a secondary analysis of data from a controlled randomized trial. Revista de salud publica (Bogota, Colombia).

[73] Price BB, Amini SB, Kappeler K. Exercise in pregnancy: effect on fitness and obstetric outcomes—a randomized trial. Medicine and science in sports and exercise. 2012; 10.1249/MSS.0b013e318267ad67. 10.1249/MSS.0b013e318267ad67 22843114

[74] Ramos JG, Bgeginski R, Opperman ML, Martins-Costa S, Delevatti R, Schuch R (2015). Effect of aerobic training in pregnant women diagnosed with gestational diabetes: a preliminary report. Pregnancy Hypertension..

[75] Seneviratne SN, Jiang Y, Derraik J, McCowan L, Parry GK, Biggs JB, et al. Effects of antenatal exercise in overweight and obese pregnant women on maternal and perinatal outcomes: a randomised controlled trial. BJOG : an international journal of obstetrics and gynaecology. 2016; 10.1111/1471-0528.13738. 10.1111/1471-0528.13738 26542419

[76] Ussher M, Lewis S, Aveyard P, Manyonda I, West R, Lewis B (2015). Physical activity for smoking cessation in pregnancy: randomised controlled trial. BMJ (Clinical research ed).

[77] Wang C, Wei Y, Zhang X, Zhang Y, Xu Q, Sun Y (2017). A randomized clinical trial of exercise during pregnancy to prevent gestational diabetes mellitus and improve pregnancy outcome in overweight and obese pregnant women. Am J Obstet Gynecol.

[78] Kihlstrand M, Stenman B, Nilsson S, Axelsson O (1999). Water-gymnastics reduced the intensity of back/low back pain in pregnant women. Acta Obstet Gynecol Scand.

[79] Ruiz JR, Perales M, Pelaez M, Lopez C, Lucia A, Barakat R (2013). Supervised exercise-based intervention to prevent excessive gestational weight gain: a randomized controlled trial. Mayo Clin Proc.

[80] Sklempe Kokic I, Ivanisevic M, Biolo G, Simunic B, Kokic T, Pisot R. Combination of a structured aerobic and resistance exercise improves glycaemic control in pregnant women diagnosed with gestational diabetes mellitus. A randomised controlled trial. Women and birth. 2017; 10.1016/j.wombi.2017.10.004. 10.1016/j.wombi.2017.10.004 29055674

[81] Santos IA, Stein R, Fuchs SC, Duncan BB, Ribeiro JP, Kroeff LR (2005). Aerobic exercise and submaximal functional capacity in overweight pregnant women: a randomized trial. Obstet Gynecol.

[82] Barakat R, Pelaez M, Montejo R, Refoyo I, Coteron J. Exercise throughout pregnancy does not cause preterm delivery: a randomized, controlled trial. J Phys Act Health. 2014; 10.1123/jpah.2012-0344. 10.1123/jpah.2012-0344 23676311

[83] Barakat R, Ruiz JR, Stirling JR, Zakynthinaki M, Lucia A. Type of delivery is not affected by light resistance and toning exercise training during pregnancy: a randomized controlled trial. Am J Obstet Gynecol. 2009; 10.1016/j.ajog.2009.06.004. 10.1016/j.ajog.2009.06.004 19608151

[84] Cavalcante SR, Cecatti JG, Pereira RI, Baciuk EP, Bernardo AL, Silveira C. Water aerobics II: maternal body composition and perinatal outcomes after a program for low risk pregnant women. Reprod Health. 2009;6(1) 10.1186/1742-4755-6-1. 10.1186/1742-4755-6-1 PMC2628875 19126239

[85] Chiavaroli V, Hopkins S, Biggs J, Derraik J, Rodrigues RO, Cutfield W et al. Regular, moderate intensity maternal exercise reduces birth weight but increases the risk of later childhood adiposity. International journal of pediatric endocrinology conference: 8th biennial scientific meeting of the asia pacific paediatric endocrine society, APPES 2014 Australia conference start: 20141029 conference end: 20141101 2017.

[86] Guelfi KJ, Ong MJ, Fournier PA, Wallman KE, Grove JR, Doherty DA (2015). Does supervised home-based exercise during pregnancy reduce the recurrence of gestational diabetes? A randomised controlled trial. Journal of Paediatrics and Child Health.

[87] Haakstad L, Bo K. Exercise during pregnancy—does it impact offspring birth weight parameters? J Sci Med Sport. 2012; 10.1016/j.jsams.2012.11.828.

[88] Hopkins SA, Baldi JC, Cutfield WS, McCowan L, Hofman PL. Effects of exercise training on maternal hormonal changes in pregnancy. Clin Endocrinol. 2011; 10.1111/j.1365-2265.2010.03964.x. 10.1111/j.1365-2265.2010.03964.x 21198740

[89] Hopkins SA, Baldi JC, Cutfield WS, McCowan L, Hofman PL (2013). Sex differences in fetal growth in response to exercise in pregnancy. Reprod Sci.

[90] Kasawara KT, Burgos CS, Costa ML, JL ES (2012). PP046. Adherence to exercise with bicycle during pregnancy in women with risk of preeclampsia. Pregnancy Hypertension..

[91] Kasawara KT, Burgos CSG, Nascimento SL, Costa ML, Surita F, Pinto ESJL (2012). Effects of exercise on maternal and neonatal outcomes in pregnant women with chronic hypertension and/or previous preecampsia: a randomized clinical trial. Pregnancy Hypertension.

[92] Kong KL, Campbell C, Wagner K, Peterson A, Lanningham-Foster L. Impact of a walking intervention during pregnancy on post-partum weight retention and infant anthropometric outcomes. J Dev Orig Health Dis. 2014; 10.1017/S2040174414000117. 10.1017/S2040174414000117 24901666

[93] Phelan S, Hart CN, Phipps MG, Abrams B, Schaffner A, Adams A (2011). Maternal weight gain and behaviors during pregnancy predict offspring weight in the "fit for delivery" study. Obesity.

[94] Ramirez-Velez R (2013). Effect of recommended physical activity dose on obstetrical, neonatal and maternal metabolic outcomes in pregnant Latina women. Ann Nutr Metab.

[95] Ramirez-Velez R, Bustamante J, Czerniczyniec A, Aguilar de Plata AC, Lores-Arnaiz S (2013). Effect of exercise training on eNOS expression, NO production and oxygen metabolism in human placenta. PLoS ONE [Electronic Resource].

[96] Seneviratne S, Parry G, Jiang Y, Mc Cowan L, Cutfield W, Rodrigues R et al. A randomized controlled trial on the effects of antenatal exercise on birth weight and neonatal body composition. International Journal of Pediatric Endocrinology Conference: 8th Biennial Scientific Meeting of the Asia Pacific Paediatric Endocrine Society, APPES. 2014(pagination).

[97] Seneviratne SN, Jiang Y, McCowan LM, Parry G, Gusso S, Rodrigues R (2017). The improve randomised controlled trial: non-weight bearing antenatal exercise in overweight and obese women increases neonatal adiposity. Endocrine reviews conference: 97th annual meeting and expo of the endocrine society. ENDO 2015 United States conference start: 20150305 conference end: 20150308.

[98] Stafne SN, Salvesen KA, Romundstad PR, Stuge B, Morkved S (2012). Does regular exercise during pregnancy influence lumbopelvic pain? A randomized controlled trial. Acta Obstet Gynecol Scand.

[99] Van Poppel M, Oostdam N, Wouters M, Eekhoff M, Van Mechelen W (2012). FitFor2: effects of an exercise training program on the incidence of gestational diabetes. J Sci Med Sport.

[100] Van Poppel MN, Oostdam N, Wouters MG, Eekhoff MM, Van Mechelen W. A training program for women at risk for gestational diabetes. Diabetes 2011;60:A636.

[101] Seneviratne SN, Jiang Y, Derraik J, McCowan L, Parry GK, Biggs JB (2016). Effects of antenatal exercise in overweight and obese pregnant women on maternal and perinatal outcomes: a randomised controlled trial. BJOG.

[102] Haakstad LA, Bø K. Exercise in pregnant women and birth weight: a randomized controlled trial. BMC pregnancy and childbirth. 2011; 10.1186/1471-2393-11-66. 10.1186/1471-2393-11-66 PMC3198740 21961534

[103] Committee A (2015). ACOG Committee opinion no. 650: physical activity and exercise during pregnancy and the postpartum period. Obstet Gynecol.

[104] Bell R, Palma S (2000). Antenatal exercise and birthweight. Aust N Z J Obstet Gynaecol.

[105] Risnes KR, Vatten LJ, Baker JL, Jameson K, Sovio U, Kajantie E (2011). Birthweight and mortality in adulthood: a systematic review and meta-analysis. Int J Epidemiol.

[106] Kramer MS, Martin RM, Bogdanovich N, Vilchuk K, Dahhou M, Oken E (2014). Is restricted fetal growth associated with later adiposity? Observational analysis of a randomized trial. Am J Clin Nutr.

[107] Whitaker RC (2004). Predicting preschooler obesity at birth: the role of maternal obesity in early pregnancy. Pediatrics.

[108] Biosca M, Rodriguez G, Ventura P, Samper MP, Labayen I, Collado MP (2011). Central adiposity in children born small and large for gestational age. Nutr Hosp.

[109] Crume TL, Scherzinger A, Stamm E, McDuffie R, Bischoff KJ, Hamman RF (2014). The long-term impact of intrauterine growth restriction in a diverse U.S. cohort of children: the EPOCH study. Obesity (Silver Spring).

[110] Dolan MS, Sorkin JD, Hoffman DJ (2007). Birth weight is inversely associated with central adipose tissue in healthy children and adolescents. Obesity (Silver Spring).

[111] Gillman MW, Rifas-Shiman S, Berkey CS, Field AE, Colditz GA (2003). Maternal gestational diabetes, birth weight, and adolescent obesity. Pediatrics.

[112] Kuhle S, Allen AC, Veugelers PJ (2010). Perinatal and childhood risk factors for overweight in a provincial sample of Canadian grade 5 students. Int J Pediatr Obes.

[113] Kuhle S, Maguire B, Ata N, MacInnis N, Dodds L (2017). Birth weight for gestational age, anthropometric measures, and cardiovascular disease markers in children. J Pediatr.

[114] Nordman H, Voutilainen R, Laitinen T, Antikainen L, Huopio H, Heinonen S (2016). Growth and cardiovascular risk factors in Prepubertal children born large or small for gestational age. Horm Res Paediatr.

[115] Perng W, Hajj H, Belfort MB, Rifas-Shiman SL, Kramer MS, Gillman MW, et al. Birth size, early life weight gain, and Midchildhood Cardiometabolic health. J Pediatr. 2016;173:122–30 e1 10.1016/j.jpeds.2016.02.053. 10.1016/j.jpeds.2016.02.053 PMC4884526 26995700

[116] Bell RJ, Palma SM, Lumley JM. The effect of vigorous exercise during pregnancy on birth-weight. Aust N Z J Obstet Gynaecol. 1995;35(1):46-51. 10.1111/j.1479-828x.1995.tb01829.x 7771999

[117] Narendran S, Nagarathna R, Gunasheela S, Nagendra HR (2005). Efficacy of yoga in pregnant women with abnormal Doppler study of umbilical and uterine arteries. J Indian Med Assoc.

[118] ACOG (2002). ACOG Committee opinion. Number 267, January 2002: exercise during pregnancy and the postpartum period. Obstet Gynecol.

[119] Davies GA, Wolfe LA, Mottola MF, MacKinnon C, Arsenault MY, Bartellas E (2003). Exercise in pregnancy and the postpartum period. Journal of obstetrics and gynaecology Canada : JOGC = Journal d'obstetrique et gynecologie du Canada : JOGC.

[120] Evenson KR, Savitz DA, Huston SL (2004). Leisure-time physical activity among pregnant women in the US. Paediatr Perinat Epidemiol.

[121] Pivarnik JM, Mudd LM, White EE, Schlaff RA, Peyer KL (2014). Physical activity during pregnancy and offspring characteristics at 8-10 years. J Sports Med Phys Fitness.

[122] Tanvig M, Vinter CA, Jorgensen JS, Wehberg S, Ovesen PG, Beck-Nielsen H (2015). Effects of lifestyle intervention in pregnancy and anthropometrics at birth on offspring metabolic profile at 2.8 years: results from the Lifestyle in Pregnancy and Offspring (LiPO) study. J Clin Endocrinol Metab.

[123] Catalano PM, Farrell K, Thomas A, Huston-Presley L, Mencin P, de Mouzon SH (2009). Perinatal risk factors for childhood obesity and metabolic dysregulation. Am J Clin Nutr.

