# Supplementary figures and images for: Exercise in Pregnancy and Children’s Cardiometabolic Risk Factors: a Systematic Review and Meta-Analysis
Source: Sports Med Open. 2018 Aug 2;4:35. doi: 10.1186/s40798-018-0148-x (PMC6070449; doi:10.1186/s40798-018-0148-x)

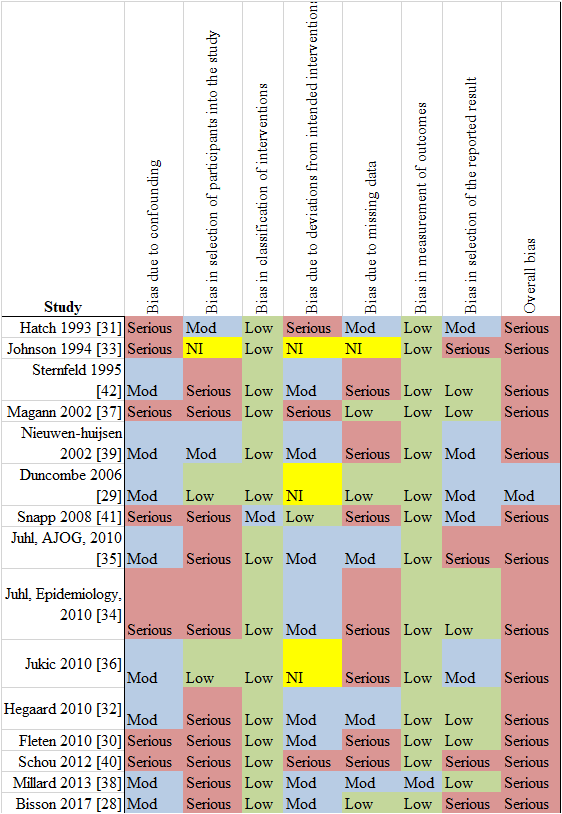

Supplement: Supplementary file 3 — Figure S3. Summary of risk of bias for individual studies following the Risk Of Bias In Non-randomized Studies of Interventions tool. The possible categories of risk of bias are: Low (green), Moderate (Mod; blue), Serious (red), Critical (gray), and No information (NI; yellow). (TIF 111 kb) [file 40798_2018_148_MOESM3_ESM.tif]
